# Observational study of frailty in older Japanese patients with non-valvular atrial fibrillation receiving anticoagulation therapy

**DOI:** 10.1038/s41598-024-65237-4

**Published:** 2024-06-22

**Authors:** Kunihiko Matsui, Kengo Kusano, Masaharu Akao, Hikari Tsuji, Shinya Hiramitsu, Yutaka Hatori, Hironori Odakura, Hisao Ogawa

**Affiliations:** 1 Department of General Medicine and Primary Care, Kumamoto University Hospital, 1-1-1 Honjyo Chuo-ku, Kumamoto, 860-8556 Japan; 2 Department of Cardiovascular Medicine, National Cerebral and Cardiovascular Center, Suita, Japan; 3 Department of Cardiology, National Hospital Organization Kyoto Medical Center, Kyoto, Japan; 4 Tsuji Clinic, Kyoto, Japan; 5 Hiramitsu Heart Clinic, Nagoya, Japan; 6 Hatori Clinic, Kawasaki, Japan; 7 Dobashi Naika Clinic, Sendai, Japan; 8 Kumamoto University, Kumamoto, Japan

**Keywords:** Non-valvular atrial fibrillation, Anticoagulant therapy, Frailty, Japanese, Cardiology, Health care

## Abstract

The number of patients with atrial fibrillation is increasing, and frailty prevalence increases with age, posing challenges for physicians in prescribing anticoagulants to such patients because of possible harm. The effects of frailty on anticoagulant therapy in older Japanese patients with nonvalvular atrial fibrillation (NVAF) are unclear. Herein, we prescribed rivaroxaban to Japanese patients with NVAF and monitored for a mean of 2.0 years. The primary endpoint was stroke or systemic embolism. The secondary endpoints were all-cause or cardiovascular death, composite endpoint, and major or non-major bleeding. Frailty was assessed using the Japanese long-term care insurance system. A multiple imputation technique was used for missing data. The propensity score (PS) was obtained to estimate the treatment effect of frailty and was used to create two PS-matched groups. Overall, 5717 older patients had NVAF (mean age: 73.9 years), 485 (8.5%) were classified as frail. After PS matching, background characteristics were well-balanced between the groups. Rivaroxaban dosages were 10 and 15 mg/day for approximately 80% and the remaining patients, respectively. Frailty was not associated with the primary endpoint or secondary endpoints. In conclusion, frailty does not affect the effectiveness or safety of rivaroxaban anticoagulant therapy in older Japanese patients with NVAF.

**Trial registration:** UMIN000019135, NCT02633982.

## Introduction

The number of patients with atrial fibrillation (AF) is increasing in this aging society^1^. Patients with non-valvular AF (NVAF) are treated with anticoagulants to prevent thromboembolic stroke. In recent years, the use of direct oral anticoagulants (DOACs) has become widespread, replacing conventional vitamin K antagonists (VKAs), and their effectiveness has been demonstrated^2,3^. In selecting DOACs versus VKAs for this population, significant evidence remains to be explored and expected^4,5^.

Frailty is the most problematic clinical condition among the growing older population^6^. The prevalence of frailty increases with age and is associated with various adverse outcomes, including falls, disability, long-term care, and death^7^. Moreover, a recent study demonstrated the relationship among the risk of ischemic stroke, cerebral small vessel disease, and frailty^8^.

Under these circumstances, anticoagulation has increasingly been considered for older patients with NVAF and frailty and is an important pillar, as indicated in the Atrial fibrillation Better Care pathway, that improves clinical outcomes for patients with AF^9^. However, the possible role of adequate anticoagulation to decrease cognitive decline for this population remains unclear^10^. The risk of stroke and bleeding increases with increasing frailty^11^. Although anticoagulation prevents thromboembolic events, the increased likelihood of bleeding events requires consideration of the balance between these conflicting effects. Its benefits and potential negative effects remain challenging for older patients with frailty. Additionally, owing to the diversity of this population, anticoagulation therapy for older individuals with frailty is not clearly defined for each condition. Moreover, as the number of older individuals with multimorbidity increases, the expected role of generalists in anticoagulant therapy becomes as crucial as that of specialists, especially in outpatient settings.

The GENeral practitioners and Embolism pRevention in NVAF patients treated with RivAroxaban: real-Life evidence (GENERAL) study was conducted by general practitioners in Japan who used rivaroxaban (a DOAC) in patients with NVAF. This study aimed to clarify whether frailty affects the effectiveness and safety of rivaroxaban anticoagulation therapy in this older Japanese population.

## Methods

This study was conducted as part of the GENERAL study. The details of the GENERAL study have been reported elsewhere^12,13^. In brief, GENERAL was a multicenter prospective observational study involving patients prescribed rivaroxaban for NVAF in daily clinical practice, specifically by ambulatory primary care physicians in Japan (UMIN000019135, NCT02633982). The patient inclusion criteria were treatment with rivaroxaban and provision of written informed consent for participation in the present study (including those treated with rivaroxaban during the registration period and those who received ablation treatment). The dosage of rivaroxaban was determined based on the patients’ renal function measured by creatinine clearance (CLcr), which was approved by the Japanese Ministry of Health and Welfare. Rivaroxaban 15 mg/day was administered to patients with CLcr ≥ 50 mL/min, and 10 mg/day was administered to patients with CLcr between 15 and 49 mL/min. However, the dosage could be changed by the physician-in-charge.

Patient data were collected through an electric data capture system with a web-based Internet system, in addition to paper form-based collection. In addition to patients’ baseline data, outcome event-related data were collected during the follow-up period until the study ended. Endpoint-related events were confirmed by the event assessment committee, which was empowered to ask the physicians in charge of each patient’s details.

### Japanese long-term care insurance classification system

This study evaluated the physical frailty status of vulnerable patients using the Japanese long-term care insurance (LTCI) system. In this system, each applicant was assessed by his/her primary care physician using a health checkup questionnaire comprising 74 items, in addition to interviews with his/her family. Subsequently, they were assessed and approved by the local government’s regional committee in a nationwide system. The Japanese LTCI system has been widely used in Japan since 2006, and the patients are classified according to disability and care requirement levels as follows: “independent,” “support levels 1 and 2,” and “long-term care levels 1, 2, 3, 4, and 5”^14^. Individuals are classified into eight groups in total, with the latter group requiring more support and/or care and having increased frailty than the other groups. The Japanese LTCI classification system was validated using Fried’s criteria for frailty. Fried’s criteria have the following three levels: “no frail,” “pre-frail,” and “frail.” Although the range of physical function of individuals considered in the frail category is wide and overlapping, the “independent” category in the Japanese LTCI system and “no frail” category in Fried’s criteria are nearly identical^14^.

### Outcomes

The outcomes of this study were consistent with those of the original study^13^. The primary endpoint was a composite of stroke (ischemic and hemorrhagic), transient ischemic attack, and systemic embolism. The secondary endpoints were all-cause death, cardiovascular death, composite (stroke, systemic embolism, acute myocardial infarction/unstable angina, and cardiovascular death), major bleeding (defined according to the International Society on Thrombosis and Hemostasis criteria), and non-major bleeding.

### Statistical analyses

Categorical variables were assessed using the chi-squared or Fisher’s exact test, as appropriate, and continuous variables were assessed using the t-test or Kruskal–Wallis test based on the distribution. We performed a propensity score (PS) analysis to estimate the treatment effect of the LTCI classification. Because some data were missing, we used a multiple imputation technique for further analysis. Subsequently, we used the PS technique to create two groups with matched scores and analyzed the outcomes. Thereafter, we analyzed each imputed dataset and combined these results using Rubin’s rule^15^.

After assessing missing baseline characteristics, we used multiple imputations to reduce bias and increase the power of the analysis, aiming to obtaining reliable results. In the imputation model, we assumed the missing data were at random such that the probability of nonresponse depends on the observed data and not on the values of the missing data. Multiple imputation was performed using Stata with the fully conditional specification method using linear regression models for continuous variables and logistic regression models for binary/ordinal variables^16^. Each missing value was imputed 10 times using the chained equation method. In this method, a single imputation is conducted during the initial fill-in stage, and the subsequent variable is imputed using the observed and imputed values from the variables that preceded them^17^. We imputed continuous variables without transformation^18^.

Baseline characteristics with and without missing data collected at study enrollment were included in the imputation model. These included demographic characteristics (age, sex, body weight, current smoking status, current alcohol consumption status, and Japanese LTCI level), clinical characteristics (CLcr, type of AF, HASBLED score), comorbid conditions/previous histories (stroke, transient ischemic attack, systemic embolism, deep venous thrombosis, pulmonary thromboembolism, peripheral artery disease, major bleeding, cardiovascular disease, myocardial infarction, heart failure, hypertension, diabetes, dyslipidemia, chronic kidney disease, liver disease, cancer, dementia medication), and management (initial dose for rivaroxaban at study enrollment, new rivaroxaban prescription, AF treatment, percutaneous coronary intervention [PCI], coronary artery bypass grafting [CABG]). Each outcome was included in the imputation model. If the patients were lost to treatment before the study ended, they were treated as censored at the final observation point, and the first 20 iterations were dropped as a burn-in for every imputation. After completion of the imputation, we used “midiagplots” command of Stata to examine if the missing data were appropriately imputed^19^. No evident problems were observed in the imputation results.

To estimate the treatment effect of the LTCI classification, accounting for the imbalance in the patient’s background characteristics, we used the PS-matching technique. PS is defined as an individual’s conditional probability of receiving exposure, rather than control, in their given characteristics. Among patients with the same PS, exposure was unrelated to confounders, such as patient characteristics. The exposed and non-exposed groups tended to have the same distribution of the measured confounders.

We divided the participants into the following two groups based on the classification results of the LTCI system: an independent group and a dependent group, with any level of support/care. The PS was calculated using a multivariate logistic regression model for each outcome of each imputed dataset, including demographic characteristics (age, sex, body weight, current smoking status, current alcohol consumption status), clinical characteristics (CLcr, type of AF, CHADS_2_ score, HASBLED score), comorbid conditions/previous histories (stroke, transient ischemic attack, systemic embolism, deep venous thrombosis, pulmonary thromboembolism, peripheral artery disease, major bleeding, cardiovascular diseases including myocardial infarction, heart failure, hypertension, diabetes, dyslipidemia, chronic kidney disease, liver disease, cancer, dementia medication), and management (initial dose for rivaroxaban at study enrollment, new rivaroxaban prescription, AF treatment, PCI, CABG). Among the covariates, CHADS_2_ score was omitted because of collinearity, as diagnosed by variance inflation factor analysis. In addition, the PS models included outcome variables, whereas the unique version of the analysis model did not include the respective outcome variable.

After obtaining the PS for each case in each dataset for an outcome, the patients in the two groups were matched using the PS in each dataset. Matching patients with a similar estimated PS creates an approximate balance for all confounders, and the difference in outcomes within groups with a similar PS provides unbiased estimates of treatment effects. The patients between the two groups were matched by the PS with “kmatch” command in Stata, with nearest-neighbor one-to-one scheme without replacement. Between matched pairs, the maximum difference in PS was allowed 0.25 of standard deviation as the caliper level.

To show the balance of the covariates for PS before and after matching, the absolute standardized mean differences for each covariate among each outcome across imputations are shown graphically. In addition, the overlap assumption of PS between the two groups was graphically confirmed using box plots for each imputed matched data point for each outcome^20^.

In each imputed and PS-matched dataset, the treatment effects of the LTCI classification for independent assignment were assessed using Cox proportional hazards models for each outcome. We also constructed models adjusted with rivaroxaban dosage and explored their effects to each outcome. The proportional hazards assumption was graphically examined to compare the observed and predicted plots for each outcome^21^.

Sensitivity analyses were performed under various conditions. First, the population was limited to the complete case cohort, and the analyses were performed with and without adjusting for baseline backgrounds for each outcome event. Individuals with missing data were excluded from these analyses. Second, the dependent group with any support and/or care was divided into three groups according to the LTCI classification, and their treatment effects are shown. These were the support level 1 or 2, long-term care level 1 or 2, and long-term care level above 3 groups. As the independent group (no LTCI classification) was the reference, similar analyses were performed as previously, with or without adjustments, for the complete and matched imputed cases for each outcome. Third, to consider the influence of unmeasured confounding factors, patients with PS < 10% and > 90% in the entire population of each imputed dataset for each outcome were trimmed, and the analyses were repeated as above for each outcome^22^.

The analysis plan for this report was originally planned as part of subgroup analyses^12^. The number of enrolled patients in the original study was derived from the results of previous studies, and sample size calculation for this study was not performed, considering imputation for missing variables and PS matching. This study was conducted in accordance with the principles of the Declaration of Helsinki and approved by the Central Institutional Review Board (Institutional Review Board of Koyasu Neurosurgical Clinic [Yokohama, Japan], reference number 16000007), which was independent of the participating trial sites. This institutional review board followed the Guidelines for Centralizing Ethical Examinations in Multi-Institutional Joint Research and Guidance on Ethical Guidelines for Medical Research Targeting Humans (issued on February 9, 2015). Written informed consent was obtained from all the patients. All significance tests were two-tailed, and P values < 0.05 were considered statistically significant. All analyses were performed using the SAS version 9.4 (SAS Institute Inc., Cary, NC, USA) or Stata/SE version 17.0 (StataCorp LLC, College Station, TX, USA) software.

## Results

The patient enrollment period was between September 2015 and March 2017. In total, 5,372 patients from 510 clinics participated in this study, and 5,717 patients were examined in the original study. The mean age was 73.9 ± 9.5 years. In this study, we enrolled the same population as that of the original study, and the follow-up period was 2.0 ± 0.5 years, with a maximum of 3 years. In this cohort, LTCI classification data were available for all participants. Among them, 5,232 (91.5%) were independent, and 485 (8.5%) had some level of support and/or care. The missing data variables varied from 0 to 35% (Table 1). Compared with the independent individuals, the dependent individuals with any level of support and/or care were more likely to be older, female, and have low body weight. In addition, they had higher risk scores, such as CHADS_2_ scores, and were more likely to have comorbidities and previous histories, including stroke events and cardiovascular diseases. However, no differences were observed in the prevalence of hypertension, diabetes, or cancer between the two groups. Regarding management, the dependent frail individuals were more likely to have previous anticoagulation therapy and a lower dose of rivaroxaban than the independent individuals, but no differences were observed for previous PCI and CABG between the two groups. After imputation, we obtained 10 sets of imputed data, and the distribution of the imputed values with the observed values seemed reasonably similar among the different imputed datasets. The participants’ backgrounds obtained from the combined results of the imputed data were similar to those of the original data before imputation (Supplementary Table S1).

**Table 1:** Baseline characteristics according to frailty status.

	Total	Independent	Dependent (with any support or care)	P-value
	N = 5717	N = 5232	N = 485	
Age (years)	73.9 ± 9.5	73.0 ± 9.3	83.5 ± 6.4	< 0.001
Missing	0 (0.0%)	0 (0.0%)	0 (0.0%)	
Sex				
Male	3,704 (64.8%)	3,500 (66.9%)	204 (42.1%)	< 0.001
Female	2,013 (35.2%)	1,732 (33.1%)	281 (57.9%)	
Missing	0 (0.0%)	0 (0.0%)	0 (0.0%)	
Body weight (kg)	62.7 ± 13.3	63.5 ± 13.2	54.1 ± 11.3	< 0.001
Missing	397 (13.89)	370 (7.07)	27 (5.57)	
Smoking				
Never	3,562 (62.3%)	3,191 (61.0%)	371 (76.5%)	< 0.001
Past	1,070 (18.7%)	1,015 (19.4%)	55 (11.3%)	
Current	526 (9.2%)	511 (9.8%)	15 (3.1%)	
Missing	559 (9.8%)	515 (9.8%)	44 (9.1%)	
Alcohol consumption				
Never	2,606 (45.6%)	2,236 (42.7%)	370 (76.3%)	< 0.001
Sometimes	1,294 (22.6%)	1,245 (23.8%)	49 (10.1%)	
Everyday	1,229 (21.5%)	1,206 (23.1%)	23 (4.7%)	
Missing	588 (10.3%)	545 (10.4%)	43 (8.9%)	
Japanese LTCI assessment				
Independent	5,232 (91.5%)	5,232 (100.0%)	0 (0.0%)	< 0.001
Support level 1	110 (1.9%)	0 (0.0%)	110 (22.7%)	
Support level 2	79 (1.4%)	0 (0.0%)	79 (16.3%)	
Long-term care level 1	114 (2.0%)	0 (0.0%)	114 (23.5%)	
Long-term care level 2	94 (1.6%)	0 (0.0%)	94 (19.4%)	
Long-term care level 3	53 (0.9%)	0 (0.0%)	53 (10.9%)	
Long-term care level 4	26 (0.5%)	0 (0.0%)	26 (5.4%)	
Long-term care level 5	9 (0.2%)	0 (0.0%)	9 (1.9%)	
Missing	0 (0.0%)	0 (0.0%)	0 (0.0%)	
Systolic blood pressure (mmHg)	127.7 ± 15.2	127.9 ± 15.1	125.0 ± 16.2	< 0.001
Missing	145 (5.07)	134 (2.56)	11 (2.27)	
Diastolic blood pressure (mmHg)	73.4 ± 11.0	73.6 ± 11.0	71.2 ± 11.3	< 0.001
Missing	147 (5.14)	136 (2.6)	11 (2.27)	
Heart rate (/min)	73.5 ± 13.3	73.4 ± 13.3	74.5 ± 13.8	0.11
Missing	978 (34.21)	920 (17.58)	58 (11.96)	
Creatinine clearance (mL/min)	66.7 ± 27.3	68.9 ± 27.2	45.3 ± 17.7	< 0.001
Missing	716 (21.01)	678 (12.96)	38 (7.84)	
Paroxysmal AF				
No	3,398 (59.4%)	3,061 (58.5%)	337 (69.5%)	< 0.001
Yes	2,311 (40.4%)	2,163 (41.3%)	148 (30.5%)	
Missing	8 (0.1%)	8 (0.2%)	0 (0.0%)	
CHADS_2_ score	2.0 (2.0–3.0)	2.0 (1.0–3.0)	3.0 (2.0–3.0)	< 0.001
Missing	8 (0.1%)	8 (0.2%)	0 (0.0%)	
HASBLED score	1.0 (1.0–2.0)	1.0 (1.0–2.0)	2.0 (1.0–3.0)	< 0.001
Missing	0 (0%)	0 (0%)	0 (0%)	
CHA2DS2VASc score	3.0 (2.0–4.0)	3.0 (2.0–4.0)	4.0 (4.0–5.0)	< 0.001
Missing	8 (0.1%)	8 (0.2%)	0 (0.0%)	
Stroke (ischemic/hemorrhagic)				
No	5,014 (87.7%)	4,706 (89.9%)	308 (63.5%)	< 0.001
Yes	689 (12.1%)	512 (9.8%)	177 (36.5%)	
Missing	14 (0.2%)	14 (0.3%)	0 (0.0%)	
Ischemic stroke				
No	5,014 (87.7%)	4,706 (89.9%)	308 (63.5%)	< 0.001
Yes	631 (11.0%)	465 (8.9%)	166 (34.2%)	
Missing	72 (1.3%)	61 (1.2%)	11 (2.3%)	
Hemorrhagic stroke				
No	5,014 (87.7%)	4,706 (89.9%)	308 (63.5%)	< 0.001
Yes	59 (1.0%)	48 (0.9%)	11 (2.3%)	
Missing	644 (11.3%)	478 (9.1%)	166 (34.2%)	
Transient ischemic attack				
No	5,599 (97.9%)	5,130 (98.1%)	469 (96.7%)	0.021
Yes	104 (1.8%)	88 (1.7%)	16 (3.3%)	
Missing	14 (0.2%)	14 (0.3%)	0 (0.0%)	
Systemic embolism				
No	5,681 (99.4%)	5,201 (99.4%)	480 (99.0%)	0.029
Yes	22 (0.4%)	17 (0.3%)	5 (1.0%)	
Missing	14 (0.2%)	14 (0.3%)	0 (0.0%)	
Deep vein thrombosis				
No	5,658 (99.0%)	5,181 (99.0%)	477 (98.4%)	0.042
Yes	45 (0.8%)	37 (0.7%)	8 (1.6%)	
Missing	14 (0.2%)	14 (0.3%)	0 (0.0%)	
Pulmonary embolism				
No	5,689 (99.5%)	5,207 (99.5%)	482 (99.4%)	0.12
Yes	14 (0.2%)	11 (0.2%)	3 (0.6%)	
Missing	14 (0.2%)	14 (0.3%)	0 (0.0%)	
Peripheral artery disease				
No	5,570 (97.4%)	5,093 (97.3%)	477 (98.4%)	0.30
Yes	133 (2.3%)	125 (2.4%)	8 (1.6%)	
Missing	14 (0.2%)	14 (0.3%)	0 (0.0%)	
Major bleeding				
No	5,657 (99.0%)	5,180 (99.0%)	477 (98.4%)	0.049
Yes	46 (0.8%)	38 (0.7%)	8 (1.6%)	
Missing	14 (0.2%)	14 (0.3%)	0 (0.0%)	
Coronary artery disease				
No	5,150 (90.1%)	4,739 (90.6%)	411 (84.7%)	< 0.001
Yes	553 (9.7%)	479 (9.2%)	74 (15.3%)	
Missing	14 (0.2%)	14 (0.3%)	0 (0.0%)	
Myocardial infarction				
No	5,502 (96.2%)	5,040 (96.3%)	462 (95.3%)	0.15
Yes	200 (3.5%)	177 (3.4%)	23 (4.7%)	
Missing	15 (0.3%)	15 (0.3%)	0 (0.0%)	
Congestive heart failure				
No	4,485 (78.5%)	4,218 (80.6%)	267 (55.1%)	< 0.001
Yes	1,218 (21.3%)	1,000 (19.1%)	218 (44.9%)	
Missing	14 (0.2%)	14 (0.3%)	0 (0.0%)	
Hypertension				
No	1,554 (27.2%)	1,423 (27.2%)	131 (27.0%)	0.52
Yes	4,149 (72.6%)	3,795 (72.5%)	354 (73.0%)	
Missing	14 (0.2%)	14 (0.3%)	0 (0.0%)	
Diabetes				
No	4,393 (76.8%)	4,024 (76.9%)	369 (76.1%)	0.46
Yes	1,310 (22.9%)	1,194 (22.8%)	116 (23.9%)	
Missing	14 (0.2%)	14 (0.3%)	0 (0.0%)	
Dyslipidemia				
No	3,146 (55.0%)	2,851 (54.5%)	295 (60.8%)	0.017
Yes	2,557 (44.7%)	2,367 (45.2%)	190 (39.2%)	
Missing	14 (0.2%)	14 (0.3%)	0 (0.0%)	
Chronic kidney disease				
No	5,033 (88.0%)	4,679 (89.4%)	354 (73.0%)	< 0.001
Yes	670 (11.7%)	539 (10.3%)	131 (27.0%)	
Missing	14 (0.2%)	14 (0.3%)	0 (0.0%)	
Liver disease				
No	5,266 (92.1%)	4,800 (91.7%)	466 (96.1%)	0.003
Yes	437 (7.6%)	418 (8.0%)	19 (3.9%)	
Missing	14 (0.2%)	14 (0.3%)	0 (0.0%)	
Cancer				
No	5,266 (92.1%)	4,820 (92.1%)	446 (92.0%)	0.49
Yes	437 (7.6%)	398 (7.6%)	39 (8.0%)	
Missing	14 (0.2%)	14 (0.3%)	0 (0.0%)	
Dementia medication				
No	5,365 (93.8%)	5,058 (96.7%)	307 (63.3%)	< 0.001
Yes	338 (5.9%)	160 (3.1%)	178 (36.7%)	
Missing	14 (0.2%)	14 (0.3%)	0 (0.0%)	
Previous anticoagulants				
No	3,015 (52.7%)	2,810 (53.7%)	205 (42.3%)	< 0.001
Yes	2,322 (40.6%)	2,079 (39.7%)	243 (50.1%)	
Missing	380 (6.6%)	343 (6.6%)	37 (7.6%)	
Rivaroxaban dose per day				
< 10 mg/day	18 (0.3%)	18 (0.3%)	0 (0.0%)	< 0.001
10 mg/day	2,814 (49.2%)	2,418 (46.2%)	396 (81.6%)	
15 mg/day	2,864 (50.1%)	2,775 (53.0%)	89 (18.4%)	
15 mg/day <	2 (0.0%)	2 (0.0%)	0 (0.0%)	
Missing	19 (0.3%)	19 (0.4%)	0 (0.0%)	
Rivaroxaban new prescription				
No	5,185 (90.7%)	4,765 (91.1%)	420 (86.6%)	0.001
Yes	532 (9.3%)	467 (8.9%)	65 (13.4%)	
Missing	0 (0.0%)	0 (0.0%)	0 (0.0%)	
Treatment for AF				
No	1,682 (29.4%)	1,514 (28.9%)	168 (34.6%)	0.007
Yes	3,975 (69.5%)	3,659 (69.9%)	316 (65.2%)	
Missing	60 (1.0%)	59 (1.1%)	1 (0.2%)	
PCI				
No	5,400 (94.5%)	4,941 (94.4%)	459 (94.6%)	0.41
Yes	298 (5.2%)	272 (5.2%)	26 (5.4%)	
Missing	19 (0.3%)	19 (0.4%)	0 (0.0%)	
CABG				
No	5625 (98.4%)	5151 (98.5%)	474 (97.7%)	0.053
Yes	73 (1.3%)	62 (1.2%)	11 (2.3%)	
Missing	19 (0.3%)	19 (0.4%)	0 (0.0%)	

*LTCI* long-term care insurance, *AF* atrial fibrillation, *PCI* percutaneous coronary intervention, *CABG* coronary artery bypass grafting.

After imputation, PS was obtained for each case, and the matching process was initiated. The number of matched cases among different outcomes varied (Supplementary Tables S2-1 and -2). The absolute standardized mean difference of the covariates for PS was well balanced after matching (Supplementary Fig. S1). The overlap assumption of the distribution PS was confirmed by the box plots.

**Figure 1:**
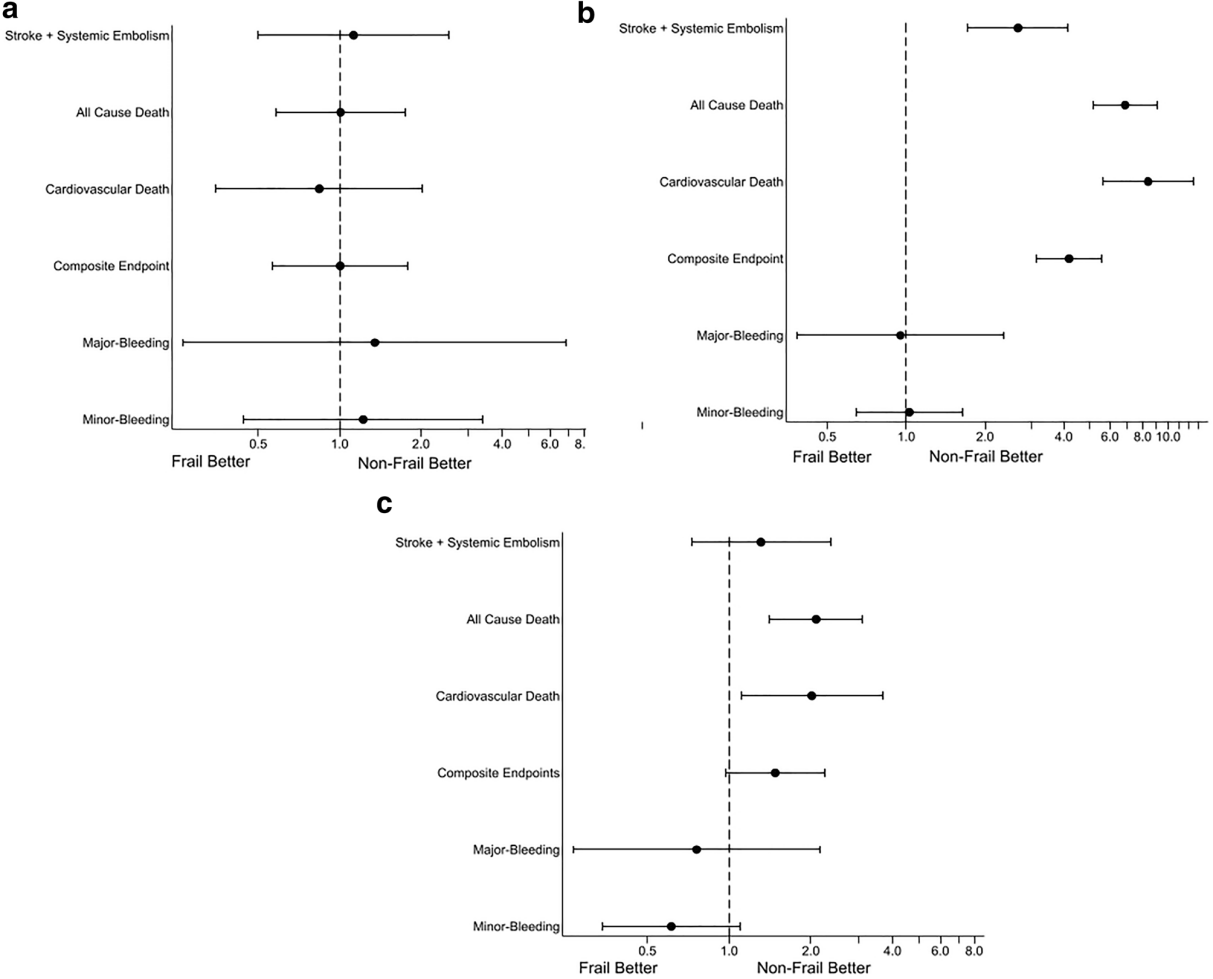
Hazard ratios and 95% confidence intervals of the treatment effects of frailty on each outcome in the different cohorts. (**a**) Imputed matched cohort. (**b**) Complete case cohort. (**c**) Complete case cohort with adjustment.

For the primary endpoints (stroke and systemic embolism), 856 patients were identified after PS matching (Supplementary Table S2-1). The treatment effect of the dependent classification of frailty was not associated with the outcome events (hazard ratio, 1.128; 95% confidence interval 0.500–2.547; P = 0.767) (Fig. 1a). Furthermore, we constructed models adjusted with rivaroxaban dosage at the study enrollment for each outcome and confirmed the similar results obtained. Similarly, for the secondary endpoints, no significant difference was consistently observed between the independent and dependent frail groups. The graphically assessed proportional hazards assumptions for each outcome were not violated.

### Sensitivity analyses

**Figure 2:**
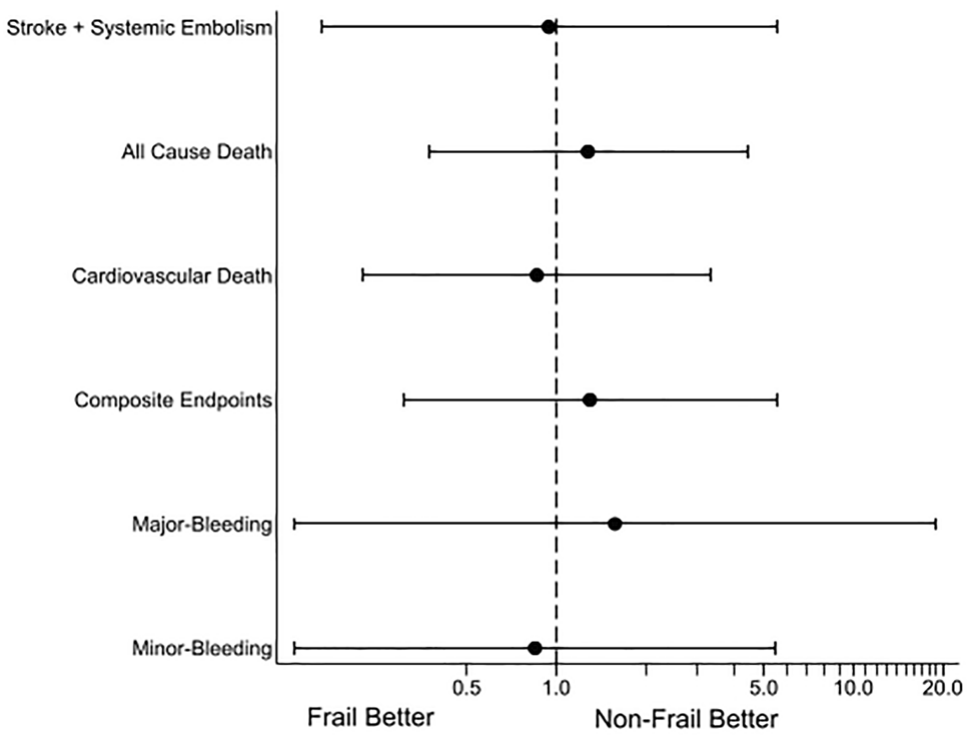
Hazard ratios and 95% confidence intervals of the treatment effects of frailty on each outcome in the trimmed imputed matched cohort.

Sensitivity analyses were performed for the different situations and populations. First, we analyzed the complete case cohort before matching with and without adjusting for clinical characteristics. Without adjustment, statistically significant treatment effects of frailty on the outcome events were observed, except for bleeding events (Fig. 1b). For the complete case cohort with adjustments, a significant effect of the treatment effects of frailty was observed for all-cause and cardiovascular deaths, but not for the other outcomes (Fig. 1c). Second, we divided the dependent group into three groups according to LTCI assessment and compared them with the independent group as a reference. Although the sample size was limited, the higher support and/or care groups tended to show higher hazard ratios in the complete case-cohort analyses, both with and without adjustments. However, these were diminished in the imputed-matched cohort for all outcomes (Supplementary Fig. S2a–c). Finally, in the trimmed case analyses, the treatment effects of frailty were not significant for all outcomes. These results were consistent with the original results of the imputed matched cohort (Fig. 2).

## Discussion

In this study, frailty did not affect the outcomes of patients with NVAF who received rivaroxaban anticoagulant therapy. Comparing outcomes based on the presence of frailty, the treatment effect on outcome events was attenuated and not observed after matching and adjusting for patient backgrounds, including age and comorbidities. As rivaroxaban was prescribed to all patients in this study, directly assessing the magnitude of its treatment effect of frailty on the outcome events was not feasible. These findings suggest that rivaroxaban is similarly effective in preventing thromboembolic events and safe in terms of bleeding events, regardless with frailty in this older Japanese population.

Several studies have reported various factors associated with frailty. For instance, the prevalence of frailty increases with age, and frailty increases the risk of death^23^. However, age is not associated with frailty diagnosis^24^. Moreover, the concept of frailty differs from that of comorbidity and disability, and different etiologies account for frailty. In light of the growing population with frailty, the use of anticoagulation therapy should be considered. An increased risk of both thromboembolic and bleeding events is observed with advanced age, and although anticoagulation is desirable owing to the high thromboembolic risk for this population, the major concern is the risk of bleeding when using anticoagulants^25^. For instance, frailty is associated with an increased risk of falling^24^. Therefore, physicians are reluctant to administer anticoagulation therapy to frail older patients. A recent systematic review of frailty and anticoagulation therapy demonstrated that frailty has led to a reluctance to prescribe anticoagulants^26^.

However, several trials have been conducted to demonstrate the efficacy and safety of anticoagulation therapies and determine optimal strategies. In recent years, anticoagulants have replaced traditional VKAs with DOACs. Although the efficacy of DOACs is comparable to that of VKAs, the safety of DOACs with respect to bleeding risk in older patients has not been clearly demonstrated until recently^27^. In a study comparing apixaban and warfarin, apixaban exhibited superior efficacy and safety, regardless of the history of falls^28^. This study also showed an increased incidence of major bleeding in patients with a history of falls, regardless of the type of anticoagulants. Additionally considering DOACs for patients with frailty, a recent study from Japan comparing low-dose edoxaban to placebo in older patients showed a slight, insignificant increase in the incidence of bleeding with edoxaban, regardless of frailty status. The study also demonstrated a reduction in the prevalence of stroke and systemic embolisms^29^. These findings underscore the effectiveness and safety of anticoagulation with DOACs, suggesting that frailty has no effect on outcome events in this population.

Notwithstanding, there remain several concerns regarding the use of anticoagulants, especially DOACs, for this population, despite their numerous clinical benefits. Moreover, it may be inappropriate to directly associate the evaluation of frailty to the decision to avoid anticoagulant use, including DOACs. Some comorbidities, particularly renal insufficiency and hepatic impairment, are more likely related to bleeding events than frailty^30^. In contrast, several issues must be considered when prescribing drugs to older patients in general, not just for anticoagulants to patients with frailty. Although it is inappropriate to prescribe a drug solely based on age^6^, older adults are prone to polypharmacy because of their multiple morbidities, which increases the risk of adverse drug events even in ambulatory care settings, as observed in this study, and requires careful consideration^31^. Older patients with increased frailty should be treated with consideration of their life expectancy, clarification of their goals, and expected benefits^32^. Anticoagulants for patients with severe frailty may not be beneficial, as expected. To reduce the risk of bleeding events, several strategies can be considered, including consideration of concomitant medications, such as antiplatelet agents. Moreover, a recent study reported that switching VKA treatment to a non-vitamin K oral anticoagulant may be associated with increased bleeding complications without reduction in thromboembolic events for frail older patients with AF^33^. Careful consideration should be applied for the choice of anticoagulants, and shared decision-making (SDM) is expected based on the current evidence, taking into account the preferences and values of patients^34^. The decision to treat patients with AF with anticoagulation requires weighing the risks and benefits of the therapy^35^. Taken together, the results of our study would be consistent with the position paper by the Society for Anticoagulation Therapy, which reported that the fear of falling from frailty was not in itself a contraindication to anticoagulation therapy^36^.

Nevertheless, our results should be interpreted with caution due to some limitations. First, our study results were obtained from a Japanese population, and the definition of frailty was based on the Japanese LTCI system. The main outcomes were obtained from dichotomized groups based on the presence of frailty. Moreover, while the “no frail” category in Fried’s criteria and “independent” category in the LTCI system are almost identical^14^, the conceptual confusion for frailty exists between several criteria and outcome measures of frailty, and some studies have considered activities of daily living as a characteristic of frailty. Although frailty instruments are closely related, considerable variations exist among the numerous instruments, resulting in conflicting study findings^37^. It should be noted that, even for this study, sufficient assurance is not guaranteed. Additionally, the frail group in our study is a combination of the “pre-frail” and “frail” categories in Fried’s criteria, and their proportions in our study were not considered. These problems may have influenced and distorted the results.

Second, the study population included patients who were considered eligible and prescribed anticoagulation therapies by their physicians. This decision is influenced by various factors, such as patient age and comorbidities, alongside frailty. Older patients with severe frailty were less likely to have been included in this study. Moreover, the used dose of rivaroxaban for this study population is low and different from the usual dose for the western patients. These concerns may have influenced the outcomes and limit the generalizability of our findings to other populations and countries.

Similarly, the size of our study populations and the observation period should be considered when interpreting the results. The original GENERAL study was a single-arm observational study, and the sample size was determined based on the results of the previous studies, including from that of the western countries^12^. However, the actual observed events in the GENERAL study were fewer than expected. Furthermore, in this study, the population size became small after PS matching. Moreover, the relatively short observation period in our study would relate to event numbers, although it was similar to that of a previous study from the western countries^38^. These factors may have caused underpowered analysis and could have led to false-negative results regarding the treatment effect of frailty and made it difficult to analyze for the details of outcomes.

Third, in addition to the previous points, we should consider the analysis processes and limitations related to them. In this study, we used multiple imputation for missing variables and the PS matching technique. The obtained results could be influenced from the detail of these processes, such as variable selection, in addition to the theoretical problems. For instance, in this study, we did not consider the possible dosage changes of rivaroxaban during the study period, in addition to the concomitant drugs, such as antiplatelets. These factors, along with some unknown unadjusted factors, could have influenced our results.

Furthermore, PS matching has faced criticisms because of increasing imbalance, inefficiency, model dependence, and bias^39^. Some unknown factors may have acted as confounders and distorted the results. Moreover, the combination of PS matching and multiple imputation is increasingly applied in practice. PS matching after multiple imputation can lead to inflated variances and over-coverage^40^. These points could have led to imprecise estimates and influence the validity and robustness of the study results. As we tried to show and confirm our analytic process, such as the balance of background factors after matching for PS, they showed good alignment. However after PS matching, we may have lost some important information with the dropped cases. Therefore, even if it seemed unlikely that the background factors that were not considered had a significant effect on our results, the consequent results from the analyses should be interpreted with caution, considering various inherent challenges.

## Conclusion

Frailty itself did not affect the effectiveness or safety of rivaroxaban anticoagulation therapy in this study including older Japanese patients. When starting anticoagulant therapy in older patients with frailty, considering the patients’ individual clinical status in addition to SDM is necessary.

### Supplementary Information

Supplementary Information.

## Data Availability

The data that support the findings of this study are available from the corresponding author (KM), upon reasonable request reviewed by the steering committee.
