# Supplementary material for: Observational study of frailty in older Japanese patients with non-valvular atrial fibrillation receiving anticoagulation therapy
Source: Sci Rep. 2024 Jun 22;14:14423. doi: 10.1038/s41598-024-65237-4 (PMC11193807; doi:10.1038/s41598-024-65237-4)
Supplement: Supplementary file 1 — Supplementary Information. [file 41598_2024_65237_MOESM1_ESM.pdf]

Supplementary Information

Observational Study of Frailty in Older Japanese Patients with Non-valvular Atrial Fibrillation Receiving Anticoagulation Therapy

Kunihiko Matsui, MD, MPH;

Kengo Kusano, MD, PhD;

Masaharu Akao, MD, PhD;

Hikari Tsuji, MD, PhD;

Shinya Hiramitsu, MD, PhD;

Yutaka Hatori, MD, PhD;

Hironori Odakura, MD, PhD;

Hisao Ogawa, MD, PhD

Supplement Tables and figures: titles and legends

Table 1S

Baseline characteristics according to frailty status after imputation for missing data

Table 2S-1

Baseline characteristics after imputation and matched data for each outcome (stroke/embolism, all-cause death, and cardiovascular death)

Table 2S-2

Baseline characteristics after imputation and matched data for each outcome (composite, major bleeding, and non-major bleeding)

Figure 1S

Balancing plots for standardized mean differences of propensity score for each outcome before and after matching

Figure 2S

Hazard ratios and 95% confidence intervals of frailty classifying the severity of each outcome in the different cohorts.

a) Imputed matched cohort

    *The confidence interval was not determined owing to the lack of cases after imputation.

b) Complete case cohort

c) Complete case cohort with adjustment

Table 1S
Baseline characteristics according to frailty status after imputation for missing data

	n = 5717	Independent n = 5232	Dependent n = 485	p value
Age ≧ 75 (years)	No	2761 (52.8 %)	46 (9.5 %)	0.000
	Yes	2471 (47.2 %)	439 (90.5 %)	
Sex	Male	3500 (66.9 %)	204 (42.1 %)	0.000
	Female	1732 (33.1 %)	281 (57.9 %)	
Body weight ≧ 50 (kg)	No	754 (14.4 %)	186 (38.3 %)	0.000
	Yes	4478 (85.6 %)	299 (61.7 %)	
Smoking	Never	3558 (68 %)	406 (83.7 %)	0.000
	Past	1112 (21.3 %)	62 (12.8 %)	
	Current	562 (10.7 %)	17 (3.4 %)	
Drinking	Never	2517 (48.1 %)	406 (83.7 %)	0.000
	Sometimes	1385 (26.5 %)	53 (11 %)	
	Everyday	1329 (25.4 %)	26 (5.3 %)	
Systolic blood pressure ≧ 140 (mmHg)	No	4148 (79.3 %)	407 (83.8 %)	0.000
	Yes	1084 (20.7 %)	78 (16.2 %)	
Creatinine Clearance (mL/min)	<30	144 (2.8 %)	76 (15.7 %)	0.000
	30 ≦, <50	1021 (19.5 %)	243 (50 %)	
	50 ≦, <80	2560 (48.9 %)	147 (30.3 %)	
	80 ≦	1507 (28.8 %)	20 (4 %)	
Paroxysmal AF	No	3066 (58.6 %)	337 (69.5 %)	0.000
	Yes	2166 (41.4 %)	148 (30.5 %)	
$CHADS_2$ score	0	169 (3.2 %)	0 (0 %)	0.000
	1	1154 (22.1 %)	35 (7.2 %)	
	2	2314 (44.2 %)	162 (33.4 %)	
	3	1255 (24 %)	175 (36.1 %)	
	4	309 (5.9 %)	99 (20.4 %)	
	5	31 (0.6 %)	14 (2.9 %)	
HASBLED score	0	632 (12.1 %)	1 (0.2 %)	0.000
	1	2652 (50.7 %)	166 (34.2 %)	
	2	1438 (27.5 %)	178 (36.7 %)	
	3	439 (8.4 %)	109 (22.5 %)	
	4	64 (1.2 %)	29 (6 %)	
	5	7 (0.1 %)	2 (0.4 %)	
CHA2DS2VASc score	0	127 (2.4 %)	0 (0 %)	0.000
	1	625 (11.9 %)	3 (0.6 %)	
	2	1169 (22.3 %)	15 (3.1 %)	
	3	1415 (27 %)	89 (18.4 %)	
	4	1186 (22.7 %)	155 (32 %)	
	5	525 (10 %)	135 (27.8 %)	
	6	157 (3 %)	65 (13.4 %)	
	7	25 (0.5 %)	20 (4.1 %)	
	8	3 (0.1 %)	3 (0.6 %)	

Stroke (Ischemic/Hemorrhagic)	No	4720 (90.2 %)	308 (63.5 %)	0.000
	Yes	512 (9.8 %)	177 (36.5 %)	
Transient ischemic attack	No	5144 (98.3 %)	469 (96.7 %)	0.019
	Yes	88 (1.7 %)	16 (3.3 %)	
Systemic embolism	No	5215 (99.7 %)	480 (99 %)	0.034
	Yes	17 (0.3 %)	5 (1 %)	
Deep vein thrombosis	No	5194 (99.3 %)	477 (98.4 %)	0.054
	Yes	38 (0.7 %)	8 (1.6 %)	
Pulmonary embolism	No	5221 (99.8 %)	482 (99.4 %)	0.110
	Yes	11 (0.2 %)	3 (0.6 %)	
Peripheral artery disease	No	5106 (97.6 %)	477 (98.4 %)	0.348
	Yes	126 (2.4 %)	8 (1.6 %)	
Major bleeding	No	5194 (99.3 %)	477 (98.4 %)	0.054
	Yes	38 (0.7 %)	8 (1.6 %)	
Coronary artery disease	No	4751 (90.8 %)	411 (84.7 %)	0.000
	Yes	481 (9.2 %)	74 (15.3 %)	
Myocardilal Infarction	No	5054 (96.6 %)	462 (95.3 %)	0.123
	Yes	178 (3.4 %)	23 (4.7 %)	
Congestive heart failure	No	4230 (80.8 %)	267 (55.1 %)	0.000
	Yes	1002 (19.2 %)	218 (44.9 %)	
Hypertension	No	1426 (27.3 %)	131 (27 %)	0.957
	Yes	3806 (72.7 %)	354 (73 %)	
Diabetes	No	4035 (77.1 %)	369 (76.1 %)	0.612
	Yes	1197 (22.9 %)	116 (23.9 %)	
Dyslipidemia	No	2858 (54.6 %)	295 (60.8 %)	0.009
	Yes	2374 (45.4 %)	190 (39.2 %)	
Chronic kidney disease	No	4693 (89.7 %)	354 (73 %)	0.000
	Yes	539 (10.3 %)	131 (27 %)	
Liver disease	No	4814 (92 %)	466 (96.1 %)	0.001
	Yes	418 (8 %)	19 (3.9 %)	

Cancer	No	4833 (92.4 %)	446 (92 %)	0.721
	Yes	399 (7.6 %)	39 (8 %)	
Dementia medication	No	5072 (96.9 %)	307 (63.3 %)	0.000
	Yes	160 (3.1 %)	178 (36.7 %)	
Rivaroxaban dosage	10mg/day	2445 (46.7 %)	396 (81.6 %)	0.000
	15mg/day	2787 (53.3 %)	89 (18.4 %)	
AF therapy	Others	1568 (30 %)	169 (34.8 %)	0.071
	Drugs	3538 (67.6 %)	307 (63.3 %)	
	Interventions	126 (2.4 %)	9 (1.9 %)	
PCI	No	4956 (94.7 %)	459 (94.6 %)	0.915
	Yes	276 (5.3 %)	26 (5.4 %)	
CABG	No	5169 (98.8 %)	474 (97.7 %)	0.057
	Yes	63 (1.2 %)	11 (2.3 %)	

AF: Atrial Fibrillation
PCI: Percutaneous Coronary Intervention
CABG: Coronary Artery Bypass Grafting

Table 2S - 1

Baseline characteristics after imputation and matched data for each outcome (Stroke/Embolism, All cause death, and Cardiovascular death)

		Stroke/Embolism (n = 856)			All cause death (n = 856)			Cardiovascular death (n = 856)		
		Independent	Dependent	p value	Independent	Dependent	p value	Independent	Dependent	p value
Age ≧ 75	No	32 (7.5 %)	45 (10.5 %)	0.151	32 (7.4 %)	45 (10.5 %)	0.151	30 (7.1 %)	45 (10.5 %)	0.090
	Yes	396 (92.5 %)	383 (89.5 %)		396 (92.6 %)	383 (89.5 %)		398 (92.9 %)	383 (89.5 %)	
Sex	Male	184 (43 %)	187 (43.7 %)	0.890	186 (43.4 %)	186 (43.5 %)	1.000	180 (41.9 %)	187 (43.7 %)	0.679
	Female	244 (57 %)	241 (56.3 %)		242 (56.6 %)	242 (56.5 %)		248 (58.1 %)	241 (56.3 %)	
Body weight ≧ 50kg	No	149 (34.9 %)	152 (35.6 %)	0.886	151 (35.4 %)	154 (36 %)	0.887	155 (36.2 %)	152 (35.5 %)	0.887
	Yes	279 (65.1 %)	276 (64.4 %)		277 (64.6 %)	274 (64 %)		273 (63.8 %)	276 (64.5 %)	
Smoking	Never	358 (83.6 %)	355 (83 %)	0.851	351 (81.9 %)	354 (82.7 %)	0.957	353 (82.5 %)	362 (84.5 %)	0.664
	Past	57 (13.4 %)	57 (13.2 %)		62 (14.5 %)	59 (13.7 %)		62 (14.5 %)	53 (12.5 %)	
	Current	13 (3 %)	16 (3.8 %)		15 (3.6 %)	15 (3.6 %)		13 (3 %)	13 (3 %)	
Drinking	Never	352 (82.2 %)	343 (80.2 %)	0.687	357 (83.4 %)	350 (81.7 %)	0.787	354 (82.8 %)	353 (82.4 %)	0.994
	Sometimes	48 (11.2 %)	56 (13.1 %)		48 (11.2 %)	51 (11.9 %)		49 (11.3 %)	50 (11.8 %)	
	Everyday	28 (6.6 %)	29 (6.7 %)		23 (5.4 %)	27 (6.3 %)		25 (5.8 %)	25 (5.8 %)	
LTCI assessment	Independent	428 (0 %)	0 (0 %)	0.000	428 (0 %)	0 (0 %)	0.000	428 (0 %)	0 (0 %)	0.000
	Support level 1	0 (0 %)	106 (24.7 %)		0 (0 %)	105 (24.5 %)		0 (0 %)	106 (24.7 %)	
	Support level 2	0 (0 %)	77 (17.9 %)		0 (0 %)	76 (17.7 %)		0 (0 %)	77 (17.9 %)	
	Long-term care level 1	0 (0 %)	103 (24.1 %)		0 (0 %)	103 (24 %)		0 (0 %)	102 (23.9 %)	
	Long-term care level 2	0 (0 %)	74 (17.3 %)		0 (0 %)	74 (17.3 %)		0 (0 %)	74 (17.3 %)	
	Long-term care level 3	0 (0 %)	38 (8.9 %)		0 (0 %)	40 (9.3 %)		0 (0 %)	39 (9.1 %)	
	Long-term care level 4	0 (0 %)	22 (5.2 %)		0 (0 %)	23 (5.3 %)		0 (0 %)	22 (5.2 %)	
	Long-term care level 5	0 (0 %)	8 (1.9 %)		0 (0 %)	8 (1.9 %)		0 (0 %)	8 (1.9 %)	
Systolic blood pressure ≧ 140 mmHg	No	343 (80.2 %)	345 (80.7 %)	0.931	350 (81.7 %)	349 (81.5 %)	1.000	350 (81.7 %)	353 (82.5 %)	0.858
	Yes	85 (19.8 %)	83 (19.3 %)		78 (18.3 %)	79 (18.5 %)		78 (18.3 %)	75 (17.5 %)	

Creatinine clearance (mL/min)	< 30	59 (13.7 %)	58 (13.6 %)	0.997	59 (13.7 %)	55 (12.9 %)	0.952	54 (12.6 %)	55 (12.8 %)	0.985
	30 ≦, < 50	216 (50.4 %)	215 (50.2 %)		212 (49.5 %)	217 (50.7 %)		216 (50.4 %)	219 (51.2 %)	
	50 ≦, < 80	137 (31.9 %)	139 (32.5 %)		140 (32.8 %)	137 (31.9 %)		144 (33.7 %)	139 (32.4 %)	
	80 ≦	17 (4 %)	16 (3.7 %)		17 (4 %)	19 (4.5 %)		14 (3.2 %)	15 (3.5 %)	
Paroxysmal AF	No	278 (65.1 %)	286 (66.9 %)	0.614	280 (65.4 %)	287 (67.1 %)	0.665	291 (67.9 %)	289 (67.6 %)	0.942
	Yes	150 (34.9 %)	142 (33.1 %)		148 (34.6 %)	141 (32.9 %)		137 (32.1 %)	139 (32.4 %)	
$CHADS_2$ score	0	0 (0 %)	0 (0 %)	0.918	0 (0 %)	0 (0 %)	0.773	0 (0 %)	0 (0 %)	0.864
	1	37 (8.6 %)	32 (7.6 %)		39 (9.1 %)	33 (7.7 %)		36 (8.5 %)	32 (7.5 %)	
	2	148 (34.5 %)	151 (35.3 %)		149 (34.7 %)	151 (35.4 %)		139 (32.4 %)	154 (36 %)	
	3	151 (35.2 %)	158 (36.8 %)		149 (34.7 %)	158 (36.9 %)		159 (37.1 %)	153 (35.7 %)	
	4	78 (18.2 %)	75 (17.6 %)		78 (18.1 %)	76 (17.7 %)		78 (18.3 %)	76 (17.8 %)	
	5	15 (3.5 %)	12 (2.7 %)		15 (3.4 %)	10 (2.3 %)		15 (3.5 %)	13 (3 %)	
Stroke (Ischemic/ Hemorrhagic)	No	285 (66.5 %)	287 (67.1 %)	0.942	293 (68.5 %)	286 (66.9 %)	0.661	294 (68.6 %)	292 (68.2 %)	0.941
	Yes	143 (33.5 %)	141 (32.9 %)		135 (31.5 %)	142 (33.1 %)		134 (31.4 %)	136 (31.8 %)	
Transient ischemic attack	No	413 (96.6 %)	413 (96.4 %)	1.000	414 (96.8 %)	414 (96.6 %)	1.000	415 (97 %)	414 (96.7 %)	1.000
	Yes	15 (3.4 %)	15 (3.6 %)		14 (3.2 %)	14 (3.4 %)		13 (3 %)	14 (3.3 %)	
Systemic embolism	No	423 (98.8 %)	423 (98.8 %)	1.000	424 (99.2 %)	424 (98.9 %)	1.000	425 (99.3 %)	424 (99.1 %)	1.000
	Yes	5 (1.2 %)	5 (1.2 %)		4 (0.8 %)	4 (1.1 %)		3 (0.7 %)	4 (0.9 %)	
Deep vein thrombosis	No	423 (98.9 %)	422 (98.5 %)	1.000	423 (98.9 %)	424 (99 %)	1.000	424 (99.2 %)	422 (98.5 %)	0.752
	Yes	5 (1.1 %)	6 (1.5 %)		5 (1.1 %)	4 (1 %)		4 (0.8 %)	6 (1.5 %)	
Pulmonary thromboembolism	No	426 (99.6 %)	426 (99.5 %)	1.000	428 (100 %)	428 (100 %)		428 (100 %)	428 (100 %)	
	Yes	2 (0.4 %)	2 (0.5 %)		0 (0 %)	0 (0 %)		0 (0 %)	0 (0 %)	
Peripheral artery disease	No	418 (97.7 %)	420 (98.1 %)	0.813	422 (98.5 %)	422 (98.7 %)	1.000	421 (98.4 %)	421 (98.3 %)	1.000
	Yes	10 (2.3 %)	8 (1.9 %)		6 (1.5 %)	6 (1.3 %)		7 (1.6 %)	7 (1.7 %)	

Major bleeding	No	420 (98.2 %)	422 (98.5 %)	0.789	421 (98.3 %)	422 (98.7 %)	1.000	420 (98.2 %)	421 (98.3 %)	1.000
	Yes	8 (1.8 %)	6 (1.5 %)		7 (1.7 %)	6 (1.3 %)		8 (1.8 %)	7 (1.7 %)	
Coronary artery disease	No	365 (85.3 %)	365 (85.3 %)	1.000	363 (84.8 %)	362 (84.5 %)	1.000	369 (86.3 %)	362 (84.5 %)	0.562
	Yes	63 (14.7 %)	63 (14.7 %)		65 (15.2 %)	66 (15.5 %)		59 (13.7 %)	66 (15.5 %)	
Myocardilal Infarction	No	396 (92.6 %)	393 (91.7 %)	0.799	393 (91.9 %)	391 (91.4 %)	0.902	399 (93.2 %)	394 (92 %)	0.601
	Yes	32 (7.4 %)	35 (8.3 %)		35 (8.1 %)	37 (8.6 %)		29 (6.8 %)	34 (8 %)	
Congestive heart failure	No	263 (61.3 %)	252 (59 %)	0.485	250 (58.4 %)	247 (57.6 %)	0.890	254 (59.3 %)	250 (58.5 %)	0.835
	Yes	165 (38.7 %)	176 (41 %)		178 (41.6 %)	181 (42.4 %)		174 (40.7 %)	178 (41.5 %)	
Hypertension	No	102 (23.8 %)	102 (23.9 %)	1.000	110 (25.6 %)	112 (26.2 %)	0.938	109 (25.6 %)	111 (25.9 %)	0.938
	Yes	326 (76.2 %)	326 (76.1 %)		318 (74.4 %)	316 (73.8 %)		319 (74.4 %)	317 (74.1 %)	
Diabetes	No	314 (73.3 %)	317 (74.1 %)	0.877	329 (76.8 %)	323 (75.5 %)	0.688	323 (75.4 %)	324 (75.7 %)	1.000
	Yes	114 (26.7 %)	111 (25.9 %)		99 (23.2 %)	105 (24.5 %)		105 (24.6 %)	104 (24.3 %)	
Dyslipidemia	No	239 (55.9 %)	243 (56.7 %)	0.836	246 (57.5 %)	249 (58.1 %)	0.890	248 (57.9 %)	245 (57.2 %)	0.890
	Yes	189 (44.1 %)	185 (43.3 %)		182 (42.5 %)	179 (41.9 %)		180 (42.1 %)	183 (42.8 %)	
Chronic kidney disease	No	325 (75.8 %)	319 (74.6 %)	0.692	331 (77.5 %)	327 (76.4 %)	0.808	329 (76.8 %)	327 (76.4 %)	0.936
	Yes	103 (24.2 %)	109 (25.4 %)		97 (22.5 %)	101 (23.6 %)		99 (23.2 %)	101 (23.6 %)	
Liver disease	No	411 (96 %)	412 (96.3 %)	1.000	410 (95.8 %)	410 (95.7 %)	1.000	407 (95.1 %)	409 (95.6 %)	0.872
	Yes	17 (4 %)	16 (3.7 %)		18 (4.2 %)	18 (4.3 %)		21 (4.9 %)	19 (4.4 %)	
Cancer	No	391 (91.3 %)	386 (90.2 %)	0.637	390 (91.2 %)	392 (91.5 %)	0.903	391 (91.3 %)	387 (90.5 %)	0.722
	Yes	37 (8.7 %)	42 (9.8 %)		38 (8.8 %)	36 (8.5 %)		37 (8.7 %)	41 (9.5 %)	
Dementia medication	No	311 (72.6 %)	312 (73 %)	1.000	312 (72.9 %)	313 (73.2 %)	1.000	308 (72 %)	315 (73.7 %)	0.645
	Yes	117 (27.4 %)	116 (27 %)		116 (27.1 %)	115 (26.8 %)		120 (28 %)	113 (26.3 %)	

Rivaroxaban dose	10mg/day	347 (81 %)	353 (82.4 %)	0.658	340 (79.5 %)	341 (79.8 %)	1.000	345 (80.6 %)	350 (81.7 %)	0.727
	15mg/day	81 (19 %)	75 (17.6 %)		88 (20.5 %)	87 (20.2 %)		83 (19.4 %)	78 (18.3 %)	
PCI	No	383 (89.5 %)	385 (90 %)	0.910	380 (88.9 %)	384 (89.7 %)	0.741	383 (89.5 %)	382 (89.2 %)	1.000
	Yes	45 (10.5 %)	43 (10 %)		48 (11.1 %)	44 (10.3 %)		45 (10.5 %)	46 (10.8 %)	
CABG	No	410 (95.9 %)	410 (95.8 %)	1.000	409 (95.5 %)	411 (95.9 %)	0.865	411 (96.1 %)	410 (95.9 %)	1.000
	Yes	18 (4.1 %)	18 (4.2 %)		19 (4.5 %)	17 (4.1 %)		17 (3.9 %)	18 (4.1 %)	
Outcome	No	408 (95.3 %)	405 (94.6 %)	0.755	381 (89 %)	365 (85.4 %)	0.125	401 (93.7 %)	398 (93.1 %)	0.784
	Yes	20 (4.7 %)	23 (5.4 %)		47 (11 %)	63 (14.6 %)		27 (6.3 %)	30 (6.9 %)	

LTCI: Long-term Care Insurance
AF: Atrial Fibrillation
PCI: Percutaneous Coronary Intervention
CABG: Coronary Artery Bypass Grafting

Total numbers may be varied due to estimation from imputation results and round off

Table 2S - 2
Baseline characteristics after imputation and matched data for each outcome (Composite, Major bleeding, and Non-major bleeding)

		Composit endpoint (n= 858)			Major bleeding (n = 860)			Non-major bleeding (n = 860)		
		Independent	Dependent	p value	Independent	Dependent	p value	Independent	Dependent	p value
Age ≧ 75	No	33 (7.8 %)	45 (10.5 %)	0.191	31 (7.2 %)	45 (10.5 %)	0.118	34 (7.8 %)	45 (10.6 %)	0.238
	Yes	396 (92 %)	384 (89.5 %)		399 (92.8 %)	385 (89.5 %)		396 (92.2 %)	385 (89.4 %)	
Sex	Male	180 (42 %)	187 (43.6 %)	0.679	180 (41.9 %)	188 (43.6 %)	0.630	181 (42.1 %)	188 (43.6 %)	0.679
	Female	249 (58 %)	242 (56.4 %)		250 (58.1 %)	242 (56.4 %)		249 (57.9 %)	242 (56.4 %)	
Body weight ≧ 50kg	No	151 (35 %)	152 (35.4 %)	1.000	159 (37 %)	154 (35.8 %)	0.777	148 (34.4 %)	152 (35.5 %)	0.830
	Yes	278 (65 %)	277 (64.6 %)		271 (63 %)	276 (64.2 %)		282 (65.6 %)	278 (64.5 %)	
Smoking	Never	353 (82 %)	353 (82.4 %)	0.662	362 (84.2 %)	359 (83.4 %)	0.954	356 (82.7 %)	351 (81.6 %)	0.654
	Past	64 (15 %)	58 (13.6 %)		53 (12.3 %)	56 (13 %)		61 (14.3 %)	62 (14.3 %)	
	Current	13 (3 %)	17 (4 %)		15 (3.5 %)	15 (3.6 %)		13 (3 %)	18 (4.1 %)	
Drinking	Never	352 (82 %)	340 (79.4 %)	0.646	355 (82.6 %)	357 (82.9 %)	0.983	355 (82.6 %)	353 (82 %)	0.983
	Sometimes	49 (11 %)	57 (13.4 %)		48 (11.1 %)	47 (11 %)		46 (10.7 %)	47 (10.9 %)	
	Everyday	29 (6.7 %)	31 (7.3 %)		27 (6.3 %)	26 (6 %)		29 (6.6 %)	30 (7.1 %)	
LTCI assessment	Independent	429 (0 %)	0 (0 %)	0.000	430 (0 %)	0 (0 %)	0.000	430 (0 %)	0 (0 %)	0.000
	Support level 1	0 (0 %)	106 (24.7 %)		0 (0 %)	106 (24.7 %)		0 (0 %)	107 (24.9 %)	
	Support level 2	0 (0 %)	76 (17.8 %)		0 (0 %)	76 (17.7 %)		0 (0 %)	76 (17.8 %)	
	Long-term care level	0 (0 %)	102 (23.9 %)		0 (0 %)	102 (23.7 %)		0 (0 %)	103 (24 %)	
	Long-term care level	0 (0 %)	75 (17.5 %)		0 (0 %)	76 (17.6 %)		0 (0 %)	76 (17.6 %)	
	Long-term care level	0 (0 %)	39 (9.1 %)		0 (0 %)	39 (9 %)		0 (0 %)	37 (8.5 %)	
	Long-term care level	0 (0 %)	22 (5.1 %)		0 (0 %)	23 (5.3 %)		0 (0 %)	23 (5.3 %)	
	Long-term care level 5	0 (0 %)	8 (1.9 %)		0 (0 %)	8 (1.9 %)		0 (0 %)	8 (1.9 %)	
Systolic blood pressure ≧ 140 mmHg	No	343 (80 %)	348 (81.1 %)	0.730	348 (80.9 %)	351 (81.7 %)	0.861	347 (80.6 %)	350 (81.4 %)	0.862
	Yes	86 (20 %)	81 (18.9 %)		82 (19.1 %)	79 (18.3 %)		83 (19.4 %)	80 (18.6 %)	
Creatinine clearance (mL/min)	< 30	55 (13 %)	56 (13 %)	0.956	55 (12.8 %)	58 (13.4 %)	0.965	59 (13.8 %)	53 (12.3 %)	0.749

	30 ≦, < 50	222 (52 %)	219 (51.1 %)		214 (49.7 %)	208 (48.4 %)		217 (50.5 %)	226 (52.5 %)	
	50 ≦, < 80	138 (32 %)	137 (32 %)		143 (33.4 %)	144 (33.4 %)		141 (32.8 %)	134 (31.3 %)	
	80 ≦	14 (3.3 %)	17 (4 %)		18 (4.1 %)	20 (4.7 %)		13 (2.9 %)	17 (3.9 %)	
Paroxysmal AF	No	291 (68 %)	292 (68.2 %)	1.000	285 (66.2 %)	294 (68.4 %)	0.561	280 (65.2 %)	293 (68.1 %)	0.386
	Yes	138 (32 %)	137 (31.8 %)		145 (33.8 %)	136 (31.6 %)		150 (34.8 %)	137 (31.9 %)	
$CHADS_2$ score	0	0 (0 %)	0 (0 %)	0.761	0 (0 %)	0 (0 %)	0.829	0 (0 %)	0 (0 %)	0.853
	1	36 (8.4 %)	32 (7.4 %)		40 (9.4 %)	33 (7.8 %)		38 (8.7 %)	32 (7.5 %)	
	2	138 (32 %)	153 (35.6 %)		148 (34.4 %)	153 (35.6 %)		146 (34 %)	153 (35.7 %)	
	3	152 (36 %)	155 (36 %)		151 (35.1 %)	157 (36.6 %)		154 (35.7 %)	158 (36.7 %)	
	4	84 (20 %)	77 (17.9 %)		75 (17.5 %)	75 (17.4 %)		77 (18 %)	76 (17.6 %)	
	5	17 (4 %)	13 (3 %)		15 (3.5 %)	11 (2.6 %)		15 (3.5 %)	11 (2.5 %)	
Stroke (Ischemic/ Hemorrhagic)	No	296 (69 %)	298 (69.5 %)	0.941	302 (70.2 %)	295 (68.7 %)	0.657	290 (67.5 %)	286 (66.5 %)	0.828
	Yes	133 (31 %)	131 (30.5 %)		128 (29.8 %)	135 (31.3 %)		140 (32.5 %)	144 (33.5 %)	
Transient ischemic attack	No	413 (96 %)	415 (96.6 %)	0.853	416 (96.7 %)	416 (96.8 %)	1.000	416 (96.7 %)	414 (96.3 %)	0.853
	Yes	16 (3.6 %)	14 (3.4 %)		14 (3.3 %)	14 (3.2 %)		14 (3.3 %)	16 (3.7 %)	
Systemic embolism	No	424 (99 %)	424 (98.8 %)	1.000	423 (98.5 %)	424 (98.5 %)	1.000	425 (98.9 %)	426 (99 %)	1.000
	Yes	5 (1.1 %)	5 (1.2 %)		7 (1.5 %)	6 (1.5 %)		5 (1.1 %)	4 (1 %)	
Deep vein thrombosis	No	424 (99 %)	423 (98.5 %)	1.000	424 (98.7 %)	426 (99 %)	0.752	424 (98.6 %)	426 (99 %)	0.752
	Yes	5 (1.1 %)	6 (1.5 %)		6 (1.3 %)	4 (1 %)		6 (1.4 %)	4 (1 %)	
Pulmonary thromoembolism	No	427 (100 %)	427 (99.6 %)	1.000	430 (100 %)	430 (100 %)		427 (99.3 %)	427 (99.3 %)	1.000
	Yes	2 (0.5 %)	2 (0.4 %)		0 (0 %)	0 (0 %)		3 (0.7 %)	3 (0.7 %)	
Peripheral artery disease	No	420 (98 %)	421 (98.1 %)	1.000	422 (98.2 %)	422 (98.1 %)	1.000	423 (98.3 %)	422 (98.2 %)	1.000
	Yes	9 (2 %)	8 (1.9 %)		8 (1.8 %)	8 (1.9 %)		7 (1.7 %)	8 (1.8 %)	
Major bleeding	No	421 (98 %)	421 (98.1 %)	1.000	423 (98.4 %)	423 (98.4 %)	1.000	423 (98.3 %)	423 (98.4 %)	1.000

	Yes	8 (1.8 %)	8 (1.9 %)		7 (1.6 %)	7 (1.6 %)		7 (1.7 %)	7 (1.6 %)	
Coronary artery disease	No	369 (86 %)	371 (86.6 %)	0.921	367 (85.3 %)	367 (85.4 %)	1.000	367 (85.4 %)	367 (85.3 %)	1.000
	Yes	60 (14 %)	58 (13.4 %)		63 (14.7 %)	63 (14.6 %)		63 (14.6 %)	63 (14.7 %)	
Myocardilal Infarction	No	395 (92 %)	395 (92 %)	1.000	396 (92.1 %)	394 (91.7 %)	0.901	395 (91.9 %)	396 (92.1 %)	1.000
	Yes	34 (7.9 %)	34 (8 %)		34 (7.9 %)	36 (8.3 %)		35 (8.1 %)	34 (7.9 %)	
Congestive heart failure	No	248 (58 %)	251 (58.6 %)	0.890	258 (59.9 %)	250 (58.2 %)	0.627	251 (58.4 %)	248 (57.6 %)	0.890
	Yes	181 (42 %)	178 (41.4 %)		172 (40.1 %)	180 (41.8 %)		179 (41.6 %)	182 (42.4 %)	
Hypertension	No	108 (25 %)	109 (25.5 %)	1.000	109 (25.3 %)	117 (27.2 %)	0.588	110 (25.5 %)	108 (25.2 %)	0.938
	Yes	321 (75 %)	320 (74.5 %)		321 (74.7 %)	313 (72.8 %)		320 (74.5 %)	322 (74.8 %)	
Diabetes	No	317 (74 %)	318 (74.1 %)	1.000	314 (73 %)	324 (75.4 %)	0.483	325 (75.5 %)	324 (75.4 %)	1.000
	Yes	112 (26 %)	111 (25.9 %)		116 (27 %)	106 (24.6 %)		105 (24.5 %)	106 (24.6 %)	
Dyslipidemia	No	250 (58 %)	253 (59.1 %)	0.890	243 (56.6 %)	252 (58.5 %)	0.581	260 (60.6 %)	254 (59 %)	0.728
	Yes	179 (42 %)	176 (40.9 %)		187 (43.4 %)	178 (41.5 %)		170 (39.4 %)	176 (41 %)	
Chronic kidney disease	No	331 (77 %)	319 (74.3 %)	0.381	332 (77.1 %)	324 (75.5 %)	0.575	336 (78 %)	328 (76.4 %)	0.569
	Yes	98 (23 %)	110 (25.7 %)		98 (22.9 %)	106 (24.5 %)		94 (22 %)	102 (23.6 %)	
Liver disease	No	412 (96 %)	411 (95.8 %)	1.000	412 (95.9 %)	413 (96 %)	1.000	412 (95.9 %)	410 (95.3 %)	0.868
	Yes	17 (4 %)	18 (4.2 %)		18 (4.1 %)	17 (4 %)		18 (4.1 %)	20 (4.7 %)	
Cancer	No	393 (92 %)	393 (91.6 %)	1.000	393 (91.3 %)	390 (90.8 %)	0.811	392 (91.2 %)	393 (91.3 %)	1.000
	Yes	36 (8.4 %)	36 (8.4 %)		37 (8.7 %)	40 (9.2 %)		38 (8.8 %)	37 (8.7 %)	
Dementia medication	No	309 (72 %)	312 (72.8 %)	0.879	315 (73.3 %)	306 (71.1 %)	0.543	315 (73.3 %)	312 (72.6 %)	0.878
	Yes	120 (28 %)	117 (27.2 %)		115 (26.7 %)	124 (28.9 %)		115 (26.7 %)	118 (27.4 %)	
Rivaroxaban dose	10mg/day	344 (80 %)	349 (81.3 %)	0.729	351 (81.6 %)	347 (80.6 %)	0.794	342 (79.4 %)	344 (80.1 %)	0.932
	15mg/day	85 (20 %)	80 (18.7 %)		79 (18.4 %)	83 (19.4 %)		88 (20.6 %)	86 (19.9 %)	

PCI	No	381 (89 %)	387 (90.3 %)	0.578	382 (88.9 %)	384 (89.3 %)	0.913	383 (89.2 %)	390 (90.6 %)	0.498
	Yes	48 (11 %)	42 (9.7 %)		48 (11.1 %)	46 (10.7 %)		47 (10.8 %)	40 (9.4 %)	
CABG	No	411 (96 %)	411 (95.7 %)	1.000	413 (96 %)	413 (96 %)	1.000	413 (96.1 %)	413 (96 %)	1.000
	Yes	18 (4.2 %)	18 (4.3 %)		17 (4 %)	17 (4 %)		17 (3.9 %)	17 (4 %)	
Outcome	No	384 (90 %)	379 (88.4 %)	0.664	422 (98.1 %)	425 (98.9 %)	0.578	398 (92.6 %)	412 (95.7 %)	0.057
	Yes	45 (10 %)	50 (11.6 %)		8 (1.9 %)	5 (1.1 %)		32 (7.4 %)	18 (4.3 %)	

LTCI: Long-term Care Insurance
AF: Atrial Fibrillation
PCI: Percutaneous Coronary Intervention
CABG: Coronary Artery Bypass Grafting

Total numbers may be varied due to estimation from imputation results and round off

Figure 1S

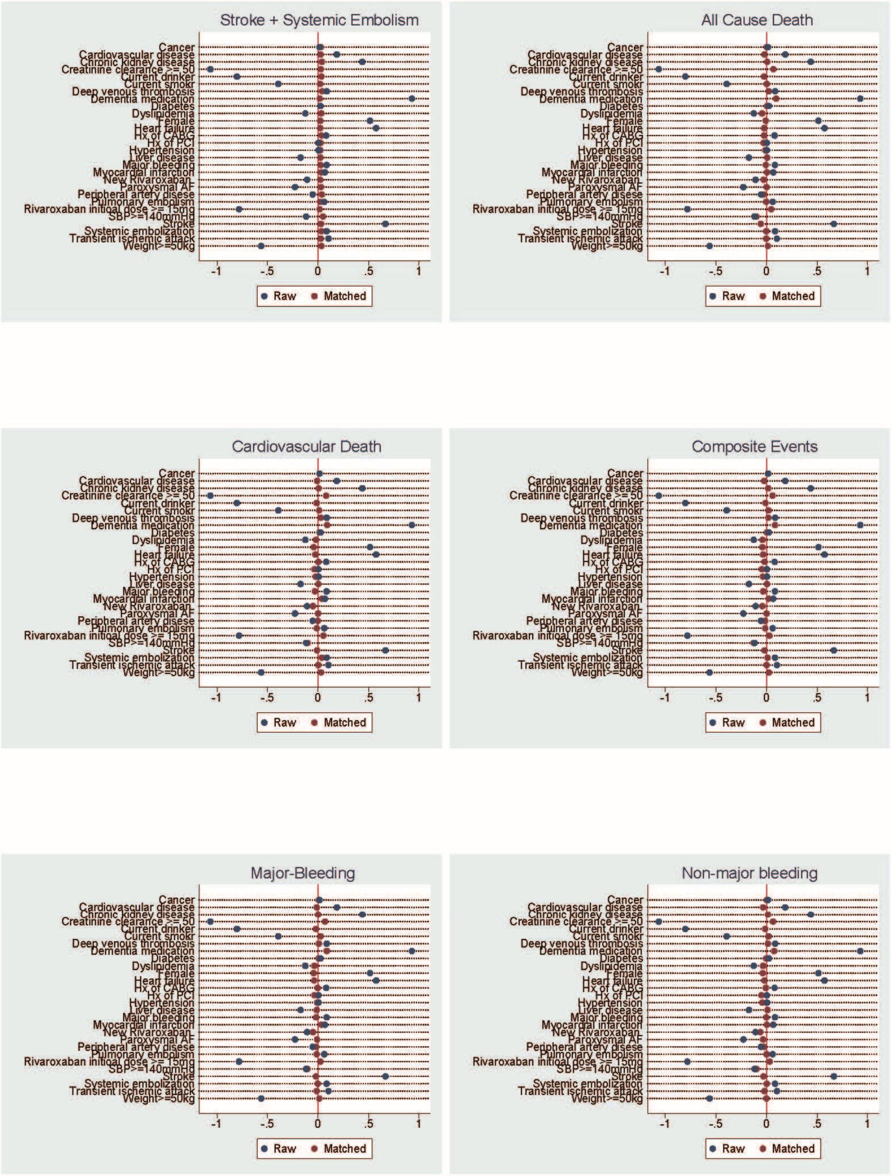

Balancing plots for standardized mean differences of propensity score for each outcome before and after matching

Figure 2S

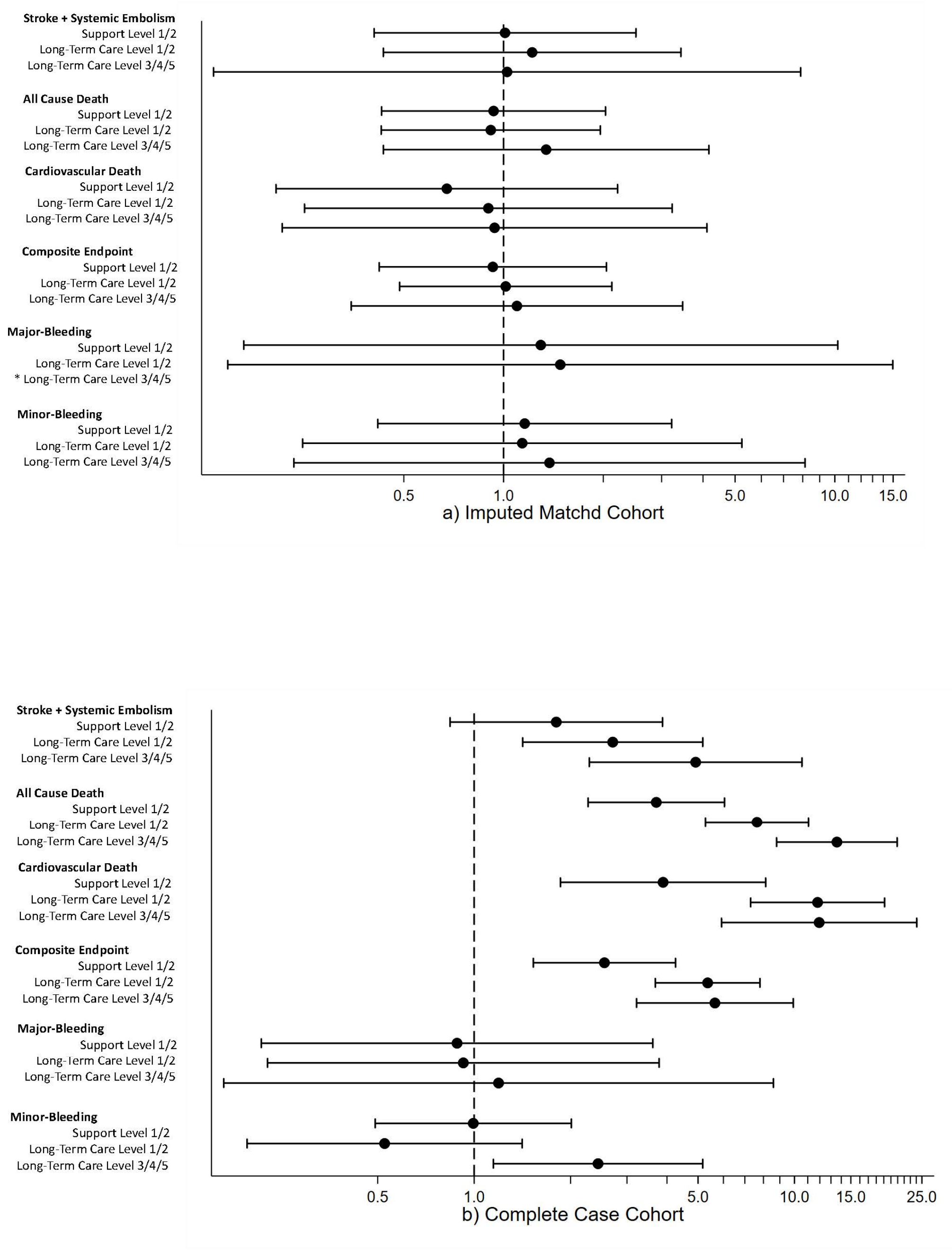
Stroke + Systemic Embolism
Support Level 1/2
Long-Term Care Level 1/2
Long-Term Care Level 3/4/5
All Cause Death
Support Level 1/2
Long-Term Care Level 1/2
Long-Term Care Level 3/4/5
Cardiovascular Death
Support Level 1/2
Long-Term Care Level 1/2
Long-Term Care Level 3/4/5
Composite Endpoint
Support Level 1/2
Long-Term Care Level 1/2
Long-Term Care Level 3/4/5
Major-Bleeding
Support Level 1/2
Long-Term Care Level 1/2
* Long-Term Care Level 3/4/5
Minor-Bleeding
Support Level 1/2
Long-Term Care Level 1/2
Long-Term Care Level 3/4/5
0.5
1.0
5.0
10.0
15.0
a) Imputed Matchd Cohort
Stroke + Systemic Embolism
Support Level 1/2
Long-Term Care Level 1/2
Long-Term Care Level 3/4/5
All Cause Death
Support Level 1/2
Long-Term Care Level 1/2
Long-Term Care Level 3/4/5
Cardiovascular Death
Support Level 1/2
Long-Term Care Level 1/2
Long-Term Care Level 3/4/5
Composite Endpoint
Support Level 1/2
Long-Term Care Level 1/2
Long-Term Care Level 3/4/5
Major-Bleeding
Support Level 1/2
Long-Term Care Level 1/2
Long-Term Care Level 3/4/5
Minor-Bleeding
Support Level 1/2
Long-Term Care Level 1/2
Long-Term Care Level 3/4/5
0.5
1.0
5.0
10.0
15.0
25.0
b) Complete Case Cohort

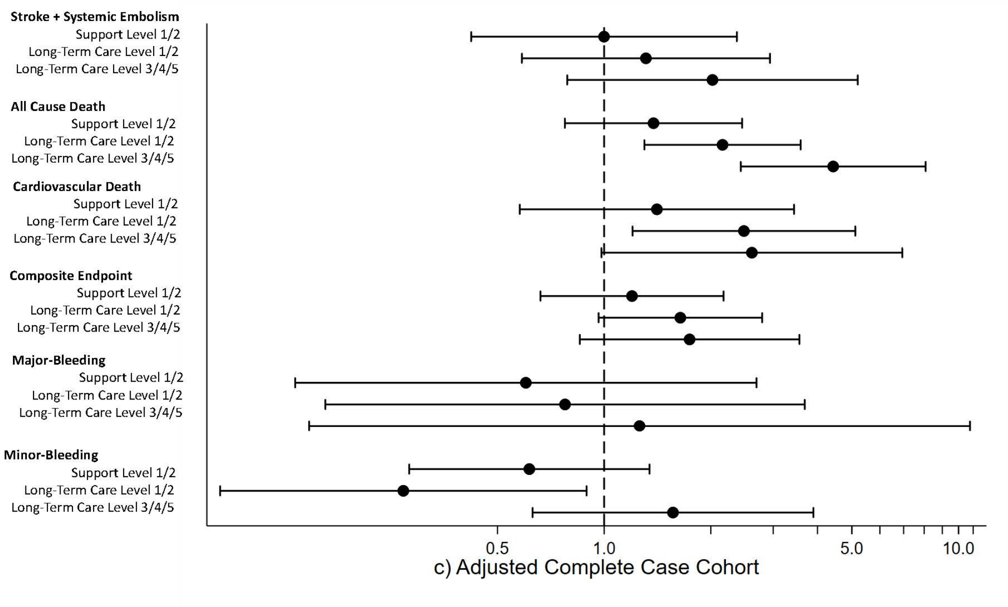

Hazard ratios and 95% confidence intervals of frailty classifying the severity of each outcome in the different cohorts.

a) Imputed matched cohort

*The confidence interval was not determined owing to the lack of cases after imputation.

b) Complete case cohort

c) Complete case cohort with adjustment
